# Post Operative Jejunal Ulcer

**Published:** 1909-10-16

**Authors:** 


### Post Operative Jejunal Ulcer.

There are few organs in the body which have so violently exercised the ingenuity of the operative surgeon as the stomach. Since the time when the first daring member of the profession, whose name unfortunately is hidden in the mists of antiquity, had the temerity to touch it, every variety of attack, frontal, lateral, posterior and combined, that experience or imagination could suggest, and manipulative dexterity or courageous perseverance carry out, has been attempted upon the organ with varying degrees of success. The literature of gastric surgery is voluminous and chaotic, and during the past ten years it has largely increased in bulk. For that very reason it is advisable that summaries of the various operative methods, their results and indications, and details of such modifications as are sanctioned by a consensus of surgical opinion, should be published from time to time. There are many interesting points in this department of surgical work that will bear investigation and research, and one of these that claims prior interest is the frequency of ulceration in the small intestine after short circuiting operations on the stomach. Hitherto this question has received comparatively little attention, and practitioners as well as specialists will therefore feel grateful to Mr. Herbert Paterson, of the Temperance Hospital, for his useful and informative monograph on "Jejunal and Gastro-jejunal Ulcer following Gastro-jejunostomy," a valuable essay that first appeared in the "Proceedings of the Royal Society of Medicine," and has now been published in book form.

Jejunal ulcer is by no means a common lesion, and the author's painstaking search through the literature has only brought to light records of sixty-three cases. That, however, is a total that can hardly be accepted as fully reliable. All cases of post operative jejunal ulcer are not diagnosed as such, nor are they published; of that, we feel sure, there can be little doubt. At the same time, it is undeniable that the lesion is an extremely rare one, and there is by no means any certitude upon the question of its ætiology. Mr. Paterson discusses the various theories of causation, the circulatory disturbance, the infective or embolic, and the hyperacidity theory in turn, without definitely declaring in favour of any one of them; and, indeed, anyone who has paid some attention to the problem of causation of these ulcers must incline to the belief that not one cause, but possibly several causative factors, are at work in producing these lesions. Hitherto the hyperacidity theory has had the field practically to itself; but, as Mr. Paterson points out, it is a theory that rests on an unphysiological assumption (if that term may correctly be used) since it presupposes that normal intestinal mucosa is capable of being acted upon by the gastric juice. That is a supposition which no physiologist will care to admit, but it is quite possible that hyperacidity may play a much larger rôle in the causation of jejunal ulcers than laboratory researches would lead us to believe. All that can be postulated at present is that there are possibly many factors at work, and that it is impossible definitely to fix the blame upon any one of them.

Mr. Paterson's conclusions are, on the whole, those with which every surgeon who has had any experience of jejunal ulcer in his own practice must concur. At the same time, we are not prepared to admit that the complication is at present less frequent than it used to be, and, as Mr. Paterson shows, the old anterior operation was not responsible for more post operative ulceration than the modern posterior one. Statistics, especially when they concern surgical work, are misleading, and certainly in this matter they are by no means clear or convincing. But there can be no doubt that the treatment must be largely preventive. The operative technique must be as faultless as possible, and there must be prolonged after treatment, while every case of recrudescent pain after gastro-jejunostomy, especially when associated with hyperacidity or hypersecretion, should be regarded as a case of potential ulcer and treated accordingly. These appear to be sound conclusions, consonant with the experience of the best surgeons of the day, and it is worth while to point out that among the operations the posterior no-loop method appears to be by far the best, since hitherto no case of ulcer has been recorded after it.

